# The Scottish Early Rheumatoid Arthritis (SERA) Study: an inception cohort and biobank

**DOI:** 10.1186/s12891-016-1318-y

**Published:** 2016-11-09

**Authors:** James Dale, Caron Paterson, Ann Tierney, Stuart H. Ralston, David M. Reid, Neil Basu, John Harvie, Neil D. McKay, Sarah Saunders, Hilary Wilson, Robin Munro, Ruth Richmond, Derek Baxter, Michael McMahon, John McLaren, Vinod Kumar, Stefan Siebert, Iain McInnes, Duncan Porter

**Affiliations:** 1 Institute of Infection, Immunity and Inflammation, College of Medical, Veterinary and Life Sciences, University of Glasgow, Glasgow, UK; 2 Wishaw General Hospital, Wishaw, UK; 3 Centre for Rheumatic Diseases, Glasgow Royal Infirmary, Glasgow, UK; 4 Institute of Genetics and Molecular Medicine, University of Edinburgh, Edinburgh, UK; 5 School of Medicine and Dentistry, University of Aberdeen, Aberdeen, UK; 6 Raigmore Hospital, Inverness, UK; 7 Western General Hospital, Edinburgh, UK; 8 Stobhill Hospital, Glasgow, UK; 9 Borders General Hospital, Melrose, UK; 10 University Hospital Ayr, Ayr, UK; 11 Dumfries and Galloway Royal Infirmary, Dumfries, UK; 12 Whyteman’s Brae Hospital, Kirkcaldy, UK; 13 Ninewell’s Hospital, Dundee, UK; 14 Gartnavel General Hospital, Glasgow, UK

## Abstract

**Background:**

The Scottish Early Rheumatoid Arthritis (SERA) study is an inception cohort of rheumatoid (RA) and undifferentiated arthritis (UA) patients that aims to provide a contemporary description of phenotype and outcome and facilitate discovery of phenotypic and prognostic biomarkers

**Methods:**

Demographic and clinical outcome data are collected from newly diagnosed RA/UA patients every 6 months from around Scotland. Health service utilization data is acquired from Information Services Division, NHS National Services Scotland. Plain radiographs of hands and feet are collected at baseline and 12 months. Additional samples of whole blood, plasma, serum and filtered urine are collected at baseline, 6 and 12 months

**Results:**

Results are available for 1073 patients; at baseline, 76 % were classified as RA and 24 % as UA. Median time from onset to first review was 163 days (IQR97-323). Methotrexate was first-line DMARD for 75 % patients. Disease activity, functional ability and health-related quality of life improved significantly between baseline and 24 months, however the proportion in any employment fell (51 to 38 %, *p* = 0.0005). 24 % patients reported symptoms of anxiety and/or depression at baseline. 35/391 (9 %) patients exhibited rapid radiographic progression after 12 months. The SERA Biobank has accrued 60,612 samples

**Conclusions:**

In routine care, newly diagnosed RA/UA patients experience significant improvements in disease activity, functional ability and health-related quality of life but have high rates of psychiatric symptoms and declining employment rates. The co-existence of a multi-domain description of phenotype and a comprehensive biobank will facilitate multi-platform translational research to identify predictive markers of phenotype and prognosis

**Electronic supplementary material:**

The online version of this article (doi:10.1186/s12891-016-1318-y) contains supplementary material, which is available to authorized users.

## Background

Rheumatoid arthritis (RA) is the commonest auto-immune inflammatory polyarthritis affecting approximately 0.8 % of the UK population [1, 2]. In the UK, an estimated 12,000 new cases of RA are diagnosed each year with an annual financial cost to the National Health Service (NHS) approaching £4billion [3, 4]. The aetio-pathogenesis of RA is complex, and incompletely understood [5]. Genetic factors account for approximately 60 % of susceptibility to the disease [6], and many single nucleotide polymorphisms and environmental factors (e.g. cigarette smoking) have been associated with an increased risk of developing RA [7, 8]. The prevailing hypothesis is that when people with a genetic susceptibility are exposed to an environmental trigger(s), tolerance to certain self-antigens is lost (sometimes as the result of citrullination of proteins) and an auto-immune response that targets the synovium (amongst other tissues) may develop [9].

In most patients, this process initially causes joint pain and stiffness and may ultimately lead to irreversible joint damage, disability and an impaired quality of life. In addition, RA patients have an increased risk of co-morbid conditions, including cardiovascular disease, infection and depression, and premature mortality [10–13]. Despite these shared features, RA is a remarkably heterogeneous disorder with a broad spectrum of disease severity, phenotype and responsiveness to treatment resulting in varying prognoses and outcomes. There is increasing evidence of genetic and molecular heterogeneity of RA: for instance, anti-citrullinated protein antibody (ACPA) positive disease is associated with different genetic (e.g. PTPN22) and environmental (e.g. smoking) risk factors when compared to ACPA-negative disease [14], and is associated with a higher rate of radiographic progression [15].

Current guidelines emphasize the need for early intensive treatment with disease modifying anti-rheumatic drugs (DMARDs) to prevent or delay disease progression [3, 16]. However, there is striking heterogeneity of treatment response and drug-related toxicity. Approximately 30 % of patients respond well to methotrexate monotherapy [17, 18], whereas others are resistant to multiple conventional and biologic DMARD therapies given singly or in combination. However, most current treatment strategies are empirical and it is not yet common practice to stratify treatment intensity based upon an estimation of prognosis. The identification of biomarkers that predict prognosis and drug responsiveness might offer the potential to characterize disease heterogeneity further, and could potentially facilitate a stratified treatment approach. Ultimately, the ability to treat the right patient with the right drug at the right time, could optimise response whilst minimizing toxicity. This hope currently rests on the findings of high throughput next generation sequencing techniques that can describe patients’ molecular phenotype (genotype, transcriptome, metabolome etc.) in unprecedented detail.

The long term course of RA appears to be changing, which may be related to the revision of the classification criteria, changing therapeutic paradigms, or alterations in the disease itself. For instance, fewer patients require major joint replacement surgery [19] and the incidence of severe extra-articular manifestations is declining [20]. Data from historic RA cohorts may therefore no longer be relevant to current RA patient populations. New, well characterised cohorts, representative of current RA patients are required to help develop and implement precision medicine as a useful clinical tool.

The Scottish Early Rheumatoid Arthritis (SERA) study was designed to create a national inception cohort of patients with newly diagnosed RA or undifferentiated arthritis (UA), capturing longitudinal phenotypic and outcome data, routine health service data, and an extensive biobank of blood and urine samples. SERA aims to provide:1. a detailed, contemporary description of the phenotype, treatment and outcome of newly diagnosed UA/RA patients in the 21st century, and 2. a resource that facilitates translational and biomarker research across multiple sequencing platforms. This report describes the study design, its Standard Operating Procedures, the cohort’s baseline characteristics and short term follow-up. It aims to raise awareness of the study’s resources within the wider rheumatology and biomarker research community in order to facilitate translational research that contributes to the delivery of individualized treatment for patients with RA.

## Methods

Rheumatology units from across Scotland participate in the SERA study. Patients with a new clinical diagnosis of RA or UA, and who have at least one swollen joint, are invited to participate. Patients are excluded if their joint swelling can be explained by an alternative diagnosis (e.g. psoriatic arthritis) or if they are carriers of blood borne viruses. Duration of symptoms up until diagnosis is not an exclusion criterion. Potential participants are referred to local SERA research nurses for screening and baseline assessments. Treatment decisions (including initiation and escalation) and clinical follow-up remain the responsibility of the local rheumatology department who follow standard local practice. Patients are not excluded if treatment with steroids or DMARDs has already started prior to recruitment (for example by the General Practitioner) as long as the diagnosis of UA/RA is new, and treatment has commenced within the last 6 months. Participants are asked to invite a first degree relative, or friend of the same gender and similar age, to participate in a cohort of healthy controls with a similar genetic or demographic background. All participants provide generic and enduring consent that allows: collection of demographic and outcome data; retrieval and linkage of routine health care data; and long term storage of data and samples for future research projects.

### Data collection

Research nurses assess participants every 6 months for two years, and annually thereafter and are responsible for assessment and collection of all of the outcome data. Baseline and follow-up demographic and clinical outcome data are collected in a standardized manner as described in Additional file 1: Table S1. Laboratory values for each visit are gathered from the hospital's laboratory records. Study records are linked to the national databases of the Information Services Division (ISD), NHS National Services Scotland using each patient’s unique Community Health Index (CHI) number. This allows the acquisition of data relating to hospital admissions (including diagnosis, operations, duration of stay), community prescription encashment, cancer diagnosis, maternal and fetal outcomes and death. Plain radiographs of both hands and both feet are collected at baseline and after 12 months follow-up.

All clinical outcome data is captured within a bespoke, online electronic case report file (eCRF) that is subject to a 6 month embargo to allow sufficient time for data entry and resolution of data queries. The presented results summarise all available data relating to clinical and radiological outcomes at baseline and over the first 24 months of follow-up in the whole cohort.

### Biological samples collection and storage

Additional blood and urine samples are collected at baseline, 6 and 12 months from virtually all patients for storage within the SERA Biobank. A detailed description of the Standard Operating Procedures for sample acquisition and storage is provided in Additional file 2. Briefly, blood samples are either stored as whole blood (PAXgene RNA and EDTA), or as 500ul aliquots of serum and plasma. If available, urine and surplus synovial fluid is also retained within the biobank. All samples are either stored locally in −80 °C freezers or are transferred immediately to the central biobank, hosted by the NHS Greater Glasgow & Clyde Bio-repository. The location and quantity of all donated biological samples is tracked using a bespoke Laboratory Information Management System (LIMS) that links directly to each participant’s electronic case report file (eCRF) and allows efficient identification of samples relating to different phenotypic subgroups

### Governance

The SERA Study was initiated by the Scottish Collaborative Arthritis Research (SCAR, www.scarnetwork.org) network and represents a collaboration between the Universities of Aberdeen, Dundee, Edinburgh and Glasgow, NHS Scotland, Healthcare Improvement Scotland, the Chief Scientist’s Office Scotland and Pfizer Ltd. The study’s protocol and procedures were reviewed and given favourable opinion by the West of Scotland Research Ethics Committee and all included patients provided written, enduring consent to participate. The study is managed by a scientific steering committee comprising clinicians and academics, from each of the participating NHS Health Boards and Universities, and (until April 2015) representatives of Pfizer Ltd. The use of samples and data is governed by the SERA Access Policy (Additional file 3) and any *bona fide* academic researcher may apply to use data and samples subject to this policy. All applications are reviewed and approved by the SERA Access Committee which comprises the scientific steering committee and four patient representatives.

## Results

Recruitment commenced in September 2011. Patients have been recruited from 16 rheumatology departments from 10 Scottish NHS Health Boards. 1073 patients had complete baseline data available in April 2015. Eighty-nine healthy controls, comprising first degree relatives or age and sex matched friends, have also been recruited. 818 (76 %) of the patients fulfilled the 2010 American College of Rheumatology (ACR)/European League Against Rheumatism (EULAR) Classification Criteria for RA [21] at the baseline assessment, and 255 (24 %) patients were classified as UA. Nineteen (7 %) of the UA patients were classified as RA during the first 2 years of follow-up. The baseline clinical and demographic features of the whole cohort are shown in Table 1. Mean age at presentation was 58 years (SD ± 14), 698 patients (65 %) are female. 772 patients (72 %) were positive for rheumatoid factor and 659 (61 %) were positive for anti-CCP antibodies. The median time from symptom onset to rheumatology referral was 115 (IQR 54–265) days and the median time from symptom onset to first rheumatology clinic review was 163 (IQR 97–323) days. Methotrexate was the first line DMARD in 810 (75 %) patients; of these 666 (82 %) received the first prescription at or after, the baseline study assessment. At the time of writing it was not possible to describe the use of DMARD or biologic therapy during the follow-up period. At the time of analysis, 830 patients had attended for 6 months assessment, 670 had attended for 12 months assessment, 378 had attended for 18 months assessment and 254 had attended for 24 months assessment.

**Table 1:** Baseline Clinical and Demographic Features

	Whole Cohort
Number	1073
Females N (%)	698 (65 %)
Age Years	58 (±14)
BMI BMI >30 N (%)	28 (±6.3) 358 (33 %)
Alcohol Excess^a^ Males N (%) Females	31 (8 %) 37 (5 %)
Current Smoker N (%)	286 (27 %)
Rheumatoid Factor Positive N (%)	772 (72 %)
Anti-CCP Antibody Positive N (%)	659 (61 %)
Symptom duration until diagnosis^b^, days	163 (IQR 97–323)
DAS28	4.74 (±1.34)
28 Swollen Joint Count	7 (±6)
28 Tender Joint Count	8 (±7)
Patient Global 100mmVAS	52 (±28)
Physician Global 100 mm VAS	45 (±24)
Pain 100 mm VAS	52 (±28)
HAQ	1.17 (±0.8)
EQ5D Index	0.51 (±0.32)
HAD Anxiety Scale ≥11 N (%)	208 (19 %)
HAD Depression Scale ≥11 N (%)	138 (13 %)
ESR^b^	22 (IQR 12–41)
CRP^b^	12 (IQR 5–33)
Commenced Methotrexate N (%)	810 (75 %)
Modified Sharp Score – Hands and Feet Erosion score^b^ Joint space narrowing score^b^ Total Sharp Score^b^	0 (IQR 0–3.0) 0 (IQR 0–4.0) 2 (IQR 0–7.0)

Values are mean (SD) unless otherwise stated

^a^Alcohol excess – defined as greater than recommended weekly intake; males >21units/week, females >14units/week

^b^Median (IQR)

**Fig. 1:**
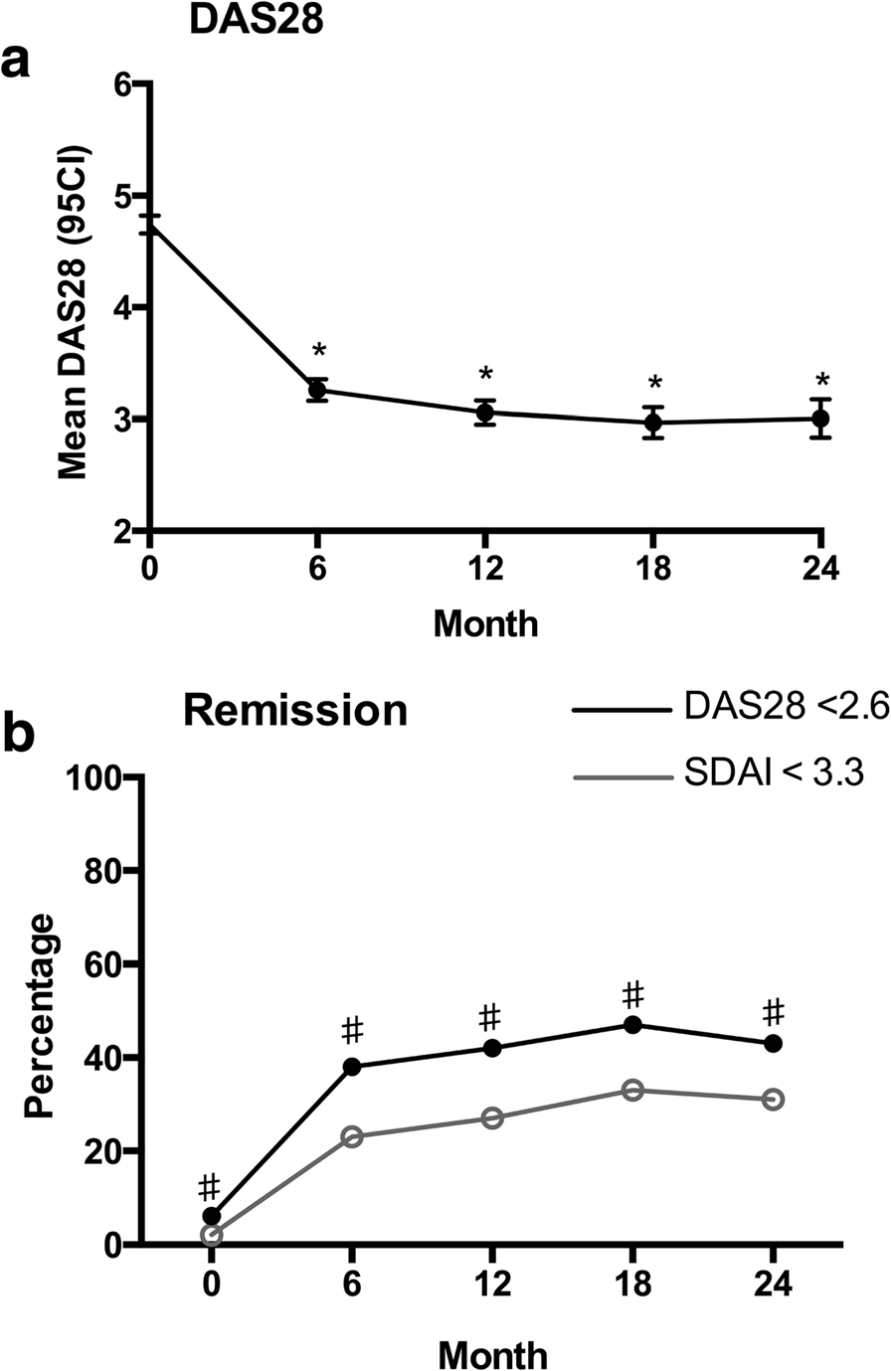
Treatment response during first 24 months of follow-up. **a**. mean DAS28, *p < 0.0001vs baseline. **b** percentage attainment of DAS28ESR and SDAI remission, ♯p < 0.05 DAS28 vs SDAI

Disease activity levels improved significantly between baseline and month 24 follow-up (Fig. 1a). Mean DAS28 fell from 4.74 (SD ± 1.34) at baseline to 3.01 (SD ± 1.40) after 24 months (*p* < 0.0001, paired *t* test). The greatest rate of improvement in DAS28 occurred between baseline and follow-up month 6. Mean change from baseline of DAS28 was −1.55 (SD ± 1.67) after 6 months, −1.70 (SD ± 1.67) after 12 months and −1.73 (SD ± 1.80) after 24 months. The proportion of patients attaining DAS28 (<2.6) and Simplified Disease Activity Index (SDAI) (<3.3) remission increased steadily over the follow-up period (Fig. 1b) with rates of DAS28 remission being significantly higher than SDAI remission at all time points. All other ACR core set variables demonstrated significant improvements from baseline during follow-up assessments (data not shown).

**Fig. 2:**
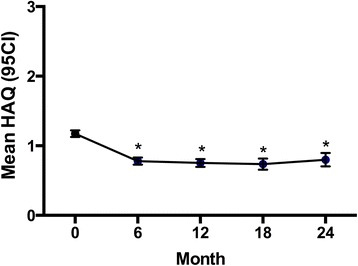
Mean HAQ during first 24 months follow-up. *p < 0.0001 vs baseline

**Fig. 3:**
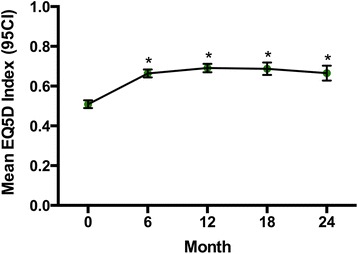
Mean EuroQoL 5D Index during first 24 months of follow-up.* *p* < 0.05 baseline vs 24 months

There were significant improvements in functional ability and health related quality of life during the follow-up period (Figs. 2 and 3). The mean Health Assessment Questionnaire (HAQ) fell from 1.17 (SD ± 0.79) at baseline to 0.80 (SD ± 0.78) after 24 months (*p* < 0.0001, paired *t* test), whereas mean EuroQol 5D index increased from 0.51 (SD ± 0.32) to 0.66 (SD ± 0.30) (*p* < 0.0001, paired *t* test)

**Fig. 4:**
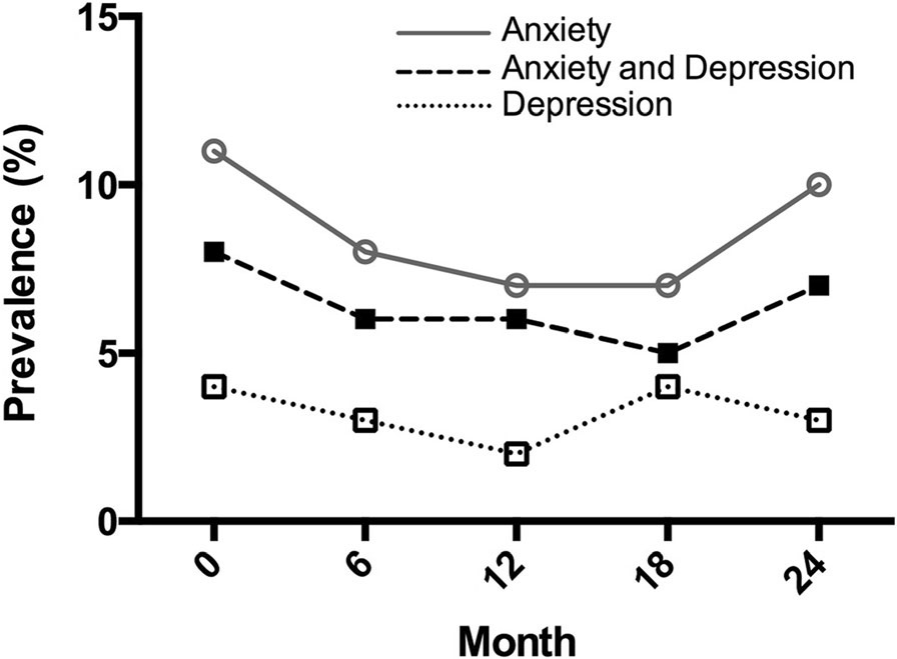
Percentage of patients fulfilling Hospital Anxiety and Depression Scale Criteria for probable anxiety (anxiety scale score ≥11), probable depression (depression scale score ≥11), probable anxiety and depression (score ≥11 on anxiety and depression scales)

At baseline, responses to the Hospital Anxiety and Depression Scale (HADS) questionnaire suggested that 23 % of patients reported symptoms of either anxiety and/or depression. Based on the HADS questionnaire responses, 11 % of patients were classified as probable anxiety (anxiety scale ≥11), 4 % as probable depression (depression scale ≥11) and 8 % with combined anxiety and depression (both scales ≥11). Furthermore, the proportion of patients reporting ongoing symptoms of anxiety, depression and a combined anxiety-depression disorder remained constant over the follow-up period (Fig. 4). Despite this, the patients’ mean emotional distress scores (total HADS) fell significantly from 11.83 (SD ± 7.9) to 9.7 (SD ± 8.2) (*p* = 0.007, paired *t* test)

**Fig. 5:**
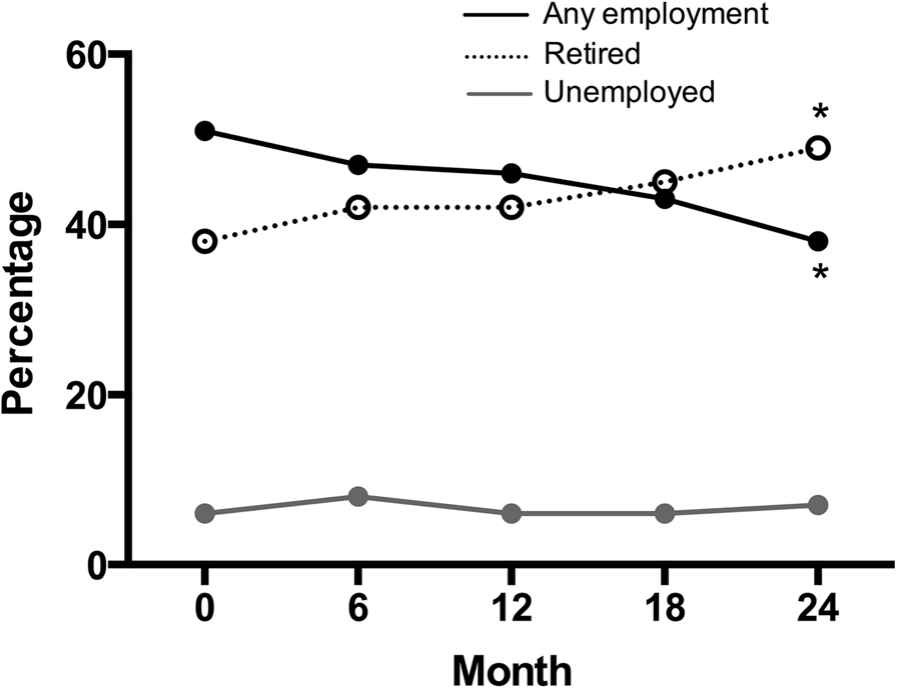
Percentage patients classified as either in any employment, unemployed or retired during first 24 months of follow-up.* *p* < 0.05, baseline vs 24 months, any employment and retired

The crude proportion of patients in any employment (full time, part time and self employed) fell significantly from 51 % at baseline to 38 % after 24 months (*p* = 0.0005, Chi square test) and was matched by a corresponding increase in the proportion of patients classified as retired (38 % at baseline, 49 % after 24 months, *p* = 0.002, CHI square test) (Fig. 5). The proportion of patients classified as unemployed remained static.

Three hundred and ninety one pairs of baseline x-rays have been graded by a private company (Imaging Rheumatology International) using the van der Heidje modified Sharp Score [22]. On baseline images, 89 (23 %) of patients had erosions scores greater than zero and 85 (22 %) had joint space narrowing scores greater than zero. After 12 months follow-up, 35 (9 %) patients demonstrated evidence of rapid radiographic progression (increase in total modified-Sharp Score >5) [23]. The median baseline values of the individual components of the modified-Sharp Score are shown in table 2.

### Additional resources

By April 2015, the SERA biobank had accrued 60,612 samples. Additional file 1: Table S2 describes the total number of aliquots available of each sample type. Thirteen research applications to access SERA resource have been approved (100 %), of which, 8 were for the use of biobank samples. Approved biobank projects have included, investigating the cohort’s DNA and RNA genotype, methotrexate pharmacogenetic profile, fine ACPA epitope reactivity, urinary proteomic signatures [24] and serum and urine metabolomics signatures.

## Discussion

The SERA cohort provides a contemporary description of the phenotype and outcome of RA/UA, as defined by the 2010 ACR-EULAR Classification Criteria, in a routine secondary-care clinical environment. In this initial description of the cohort, during the first 2 years of treatment significant improvements in measures of disease activity, functional ability and health related quality of life were observed However, there were high rates of ongoing psychiatric morbidity and a steady decline in employment.

Recent international guidelines recommend a treat-to-target approach that aims to achieve low disease activity or remission in all patients [25, 26]. However, the SERA study demonstrates that routine care of early RA in the NHS in Scotland only attains SDAI remission in a minority of cases (27 % after 12 months and 31 % after 24 months). DAS28 remission was achieved more frequently (42 % after 12 months) and compared favorably with the results from the UK-based Early RA Study (DAS28 remission 21 % after 2 years) [27]. However, DAS28 remission rates were lower than those observed in the Dutch Rheumatoid Arthritis Monitoring (DREAM) Remission Induction Cohort Study (DAS28 remission 58 % after 12 months), which used an aggressive step up strategy with early biologic use [28], and the Tight Control of RA (TICORA) study (DAS remission 62 % after 18 months) [29]. The lower remission rates observed in SERA, when compared to DREAM and TICORA, could reflect differences in the study populations, failure to rigorously implement treat-to-target principles in routine care, or potentially the more stringent eligibility criteria for biologic therapy in the UK.

The time to initiation of DMARD is an important predictor of treatment response [30] and the results of the SERA study demonstrate that Scottish rheumatologists are seeing patients at an earlier stage in their disease course than previously. In 2005–8, both phases of the Clinical Audit of Care in Rheumatoid Arthritis (CARA) in Scotland, demonstrated that the median time from symptom onset to first rheumatology review was between 245 and 308 days [31] whereas in the SERA study it was 163 days. Nonetheless, only 34 % of patients were seen within 12 weeks of symptom onset, and a notable minority of patients (23 %) already had radiographic damage at their baseline assessment.

The SERA dataset also describes how other aspects of patients’ lives may change following the diagnosis of RA. The proportion of patients who remained in any employment fell after diagnosis, even though there were significant improvements in disease activity, functional ability and health related quality of life observed over the same time period. The employment rate changes observed in the SERA cohort are similar to a recently published analysis of another UK-based early RA cohort that demonstrated that the greatest risk of work instability was within the first 2 years after diagnosis [32]. Furthermore, the results demonstrate that a significant proportion of patients (23 % at baseline) report symptoms that are consistent with anxiety and/or depression, which remained static during follow-up. These findings are similar to previous studies that used the HADS scale to describe the prevalence of anxiety and/or depression in early and established RA [33]. Taken together, these results emphasise that the diagnosis of RA can have a complex impact on many different physical, psychological and societal domains that may not be adequately addressed through solely pursuing DMARD-centric treatment strategies that focus on the eradication of inflammatory disease activity.

The size of the SERA study, the combination of careful characterization of clinical phenotype, longitudinal follow-up of outcomes and extensive biobank are significant strengths and unique for an early RA cohort. Furthermore, the ability to link to health service utilization and prescription records has the potential for the dataset to be used in a wide variety of longitudinal epidemiology projects. The availability of samples from healthy control patients is an important strength that will facilitate the identification of disease related signatures; however, it is worth highlighting the comparatively low number of healthy controls compared to patients (89 vs 1073). There is significant phenotypic overlap between RA and UA, therefore we chose to initially analyse the outcomes as a combined cohort, since in practice many clinicians treat both conditions based upon the same principles, though this may have skewed the findings. A previous study suggests that the DAS28 may be a valid outcome measure in UA [34], but the performance of the SDAI has not yet been validated. The study also has other limitations: as a research study, the patients that agree to participate may differ from those that decline; patients were recruited soon after diagnosis but nonetheless baseline clinical and laboratory findings may already have been altered by prior treatment in primary care. Multiple research nurses are employed throughout Scotland, which may introduce variability in recording outcome assessments (e.g. joint counts) and sample transfer time to the biobank. Furthermore, storage of biobank samples at −80 °C makes them unsuitable for studies that require fresh blood.

Participants continue to be assessed by research nurses, therefore the quantity of data available at each assessment time point, and the duration of follow-up, continues to increase. As the inception cohort and biobank matures the developing datasets will provide a very valuable tool for future clinical, epidemiological and systems biology research. Each dataset is available for analysis in isolation; however, it is hoped that the availability of simultaneous clinical outcome data, health records data and multiple sample types will encourage the conduct of an integrated, systems biology analysis across multiple “omic platforms” that will increase the likelihood of identifying clinically relevant and reliable predictive signatures. All of the resources described herein are available for use by *bone fide* researchers to facilitate further RA research. Details on the application procedure to access either clinical data, and/or stored biobank samples, are available on the SCAR website (www.scarnetwork.org).

## Additional files

Additional file 1: Table S1. Baseline and Clinical Follow-up Data Collection. **Table S2.** SERA Biobank. (DOC 49 kb)
Additional file 2: SERA Sample Handling SOP. (DOCX 100 kb)
Additional file 3: SERA Access Policy Holdings. (DOC 46 kb)
