# Supplementary material for: The Scottish Early Rheumatoid Arthritis (SERA) Study: an inception cohort and biobank
Source: BMC Musculoskelet Disord. 2016 Nov 9;17:461. doi: 10.1186/s12891-016-1318-y (PMC5103386; doi:10.1186/s12891-016-1318-y)
Supplement: Additional file 1: Table S1. — Baseline and Clinical Follow-up Data Collection. Table S2. SERA Biobank. (DOC 49 kb) [file 12891_2016_1318_MOESM1_ESM.doc]

### The Scottish Early Rheumatoid Arthritis (SERA) Study: an inception cohort and biobank

### Supplementary Tables

### Supplementary Table 1 – Baseline and follow-up clinical data collection

|  | - **Baseline** | - **Follow-Up Visits** |
| --- | --- | --- |
| - Symptom duration - Referral and assessment date - Past medical history - Medications - Social deprivation score - Hospital Admissions - Demographics – age, gender, marital status - Smoking and alcohol history | - ✓ - ✓ - ✓ - ✓ - ✓ | ✓  ✓ |
| - 2010 ACR-EULAR Classification Criteria - Leiden Prediction Score | - ✓ - ✓ | - ✓ |
| - DAS28 | - ✓ | - ✓ |
| - 28 Swollen Joint Count | - ✓ | - ✓ |
| - 28 Tender Joint Count | - ✓ | - ✓ |
| - Patient Global VAS | - ✓ | - ✓ |
| - Assessor Global VAS | - ✓ | - ✓ |
| - Pain VAS | - ✓ | - ✓ |
| - HAQ Score | - ✓ | - ✓ |
| - EQ5-D Questionnaire + VAS | - ✓ | - ✓ |
| - HAD Scale | - ✓ | - ✓ |
| - Fatigue - Full blood count - Urea/creatinine | - ✓ - ✓ - ✓ | - ✓ - ✓ - ✓ |
| - ESR | - ✓ | - ✓ |
| - CRP | - ✓ | - ✓ |
| - Total cholesterol | - ✓ |  |
| - HDL cholesterol | - ✓ |  |
| - Rheumatoid factor | - ✓ |  |
| - Anti-CCP antibodies | - ✓ |  |
| - X-ray Hands and Feet | - ✓ | - 12 months only |
| - Employment Status | - ✓ | - ✓ |

- DAS28 – 28 joint disease activity score, VAS – visual analogue score, HAQ – Health Assessment Questionnaire, EQ5-D – EuroQoL-5D questionnaire, HAD – Hospital Anxiety and Depression Scale, ESR – erythrocyte sedimentation rate, CRP – C-reactive protein, HDL – high density lipoprotein, anti-CCP – anti-cyclic citrillunated peptide antibodies
- **Supplementary Table 2** – SERA Biobank Holdings – correct as of April 2015

|  | - **Total number of samples** |
| --- | --- |
| - **Plasma* – EDTA** | - 10177 |
| - **Plasma* – LiHep** | 10013 |
| - **Plasma* – BDP100** | - 11330 |
| - **Serum* – SST** | - 9213 |
| - **Buffy Coat – EDTA** | - 2612 |
| - **Whole blood – EDTA** | - 2138 |
| - **Whole blood – Paxgene RNA** | - 5160 |
| - **Urine** | - 8578 |
| - **Synovial Fluid** | - 221 |
| - **DNA – FTA paper** | - 1172 |
| - **Total** | - 60612 |

- * 500ul aliquots
