# Supplementary material for: The Scottish Early Rheumatoid Arthritis (SERA) Study: an inception cohort and biobank
Source: BMC Musculoskelet Disord. 2016 Nov 9;17:461. doi: 10.1186/s12891-016-1318-y (PMC5103386; doi:10.1186/s12891-016-1318-y)
Supplement: Additional file 2: — SERA Sample Handling SOP. (DOCX 100 kb) [file 12891_2016_1318_MOESM2_ESM.docx]

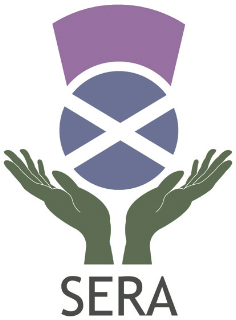


**Sample Collection, Processing, Storage and Shipping of**

**Biomarker Samples for SERA Biobank**

**Standard Operating Procedure (SOP)**

**Written by: Ashley Gilmour**

**Authorised by: Caron Paterson**

**Signed:** **Date**:

**Table of Contents**

1. SUMMARY 3

2. SAMPLE COLLECTION 3

2.1 Sample collection (SERA patient) – summary 3

2.1.1 Sample collection to be taken at baseline (T0) 3

2.1.2 Sample collection to be taken at 6 and 12 months 3

2.2 Sample tubes 3

2.2.1 Instructions on sample collection 4

2.2.2 Blood draw order 5

2.2.3 Collection of mid-stream specimen of urine 5

2.3 Sample labeling 6

2.3.1 Bar codes 6

2.4 Precautions 6

2.4.1 PAXgene RNA tubes 6

2.4.2 Urine Collection Pots 6

3. PRELIMINARY SAMPLE PROCESSING ON-SITE 6

3.1 Equipment list 6

3.2 Preliminary processing procedures 6

3.2.1 SST tubes 7

3.2.2 Lithium Heparin, 6ml EDTA and BDP100 tubes 7

3.2.3 PAXgene RNA 7

3.2.4 10ml EDTA tube for DNA 8

3.2.5 QIAcard FTA One Spot 8

3.2.6 Urine Specimen 8

4. DISPATCH OF SAMPLES TO CENTRALISED LAB 8

4.1 Transportation (see Appendix 1) 8

4.1.1 Research Nurse (within 2 hours) 8

4.1.2 Research Nurse (same day, but >2 hours) 9

4.1.3 Commercial transport – chilled, same day 9

4.1.4 Commercial transport – frozen (on dry ice) in batches every month 9

4.1.4 Sample Record log 9

5. SAMPLE PROCESSING AT CENTRAL LABORATORIES 10

6. DEPARTURES FROM SOP 10

# SUMMARY

Samples will be collected at three time points (0, 6 and 12 months) in all SERA patients according to Standard Operating Procedures (SOP) which detail the samples to be collected, the preliminary processing and storage temperatures, the transport of samples to a central processing facility, and the processing, aliquoting and storage of each sample. There should be no more than 24 hrs between the time of sample collection and the end of full sample processing. As different handling and processing techniques can generate a different type of specimen for analysis, obtaining homogenous samples is imperative. Sample collection, processing, transportation, storage and handling are as critical as the extraction and purification methods used by the down-stream user.

# SAMPLE COLLECTION

## 2.1 Sample collection (SERA patient) – summary

### 2.1.1 Sample collection to be taken at baseline (T0)

1 x 5ml SST Tube for serum

1 x 6ml Heparin/Lithium Tube for plasma

1 x 6ml EDTA Tube for plasma

1 x 10ml EDTA Tube for DNA

1 x 10ml BDP100 Tube for plasma

2 x 2.5ml PAXgene tube for RNA

Qiagen FTA card

Mid stream specimen of urine

### 2.1.2 Sample collection to be taken at 6 and 12 months

1 x 5ml SST Tube for serum

1 x 6ml Heparin/Lithium Tube for plasma

1 x 6ml EDTA Tube for plasma

1 x 10ml BDP100 Tube for plasma

2 x 2.5ml PAXgene tube for RNA

Mid stream specimen of urine

**2.1.3 Collection of synovial fluid at any scheduled or unscheduled visit**

1 x universal container with lithium heparin (or two 9ml lithium heparin Vacutainer tubes)

## 2.2 Sample tubes

SST Tube - BD Vacutainer® SST™ II Advance Tube Cat # 367954

Heparin/LithiumTube - BD Vacutainer® Plasma Tube (Heparin) Cat # 367885

6 ml EDTA Tube - BD Vacutainer® K2EDTA Tube, Cat # 367873

10 ml EDTA Tube - BD Vacutainer® K2EDTA Tube, Cat # 367525

P100 Tube - BD™ P100 Tube Cat # 366456

PAXgene RNA tube - PAXgene blood RNA tube Cat #762165

Urine -BD Urine Vacutainer Cat #

### 2.2.1 Instructions on sample collection

Sample collection boxes and sample packs will be provided with the required tubes, pipettes, sample collection record sheet, preprinted barcode labels, UN3373 and biorepository address labels. Barcode labels are to be attached to sample tubes and the Qiagen FTA card upon sample collection using the following convention.

| Table 1 – Sample labeling schema | | |
| --- | --- | --- |
| **LABEL PREFIX** | **APPLY LABEL TO:** | **APPLY SECOND LABEL TO:** |
| A | SAMPLE RECORD LOG | Retain for nurses own records |
| B | SST PARENT TUBE (YELLOW TOP) | - |
| C | LITHIUM HEPARIN PARENT TUBE (GREEN TOP) | - |
| D | 6ML EDTA PARENT TUBE (PURPLE TOP) | - |
| E | 10ML EDTA (PURPLE TOP) | - |
| F | BDP100 VACUTAINER TUBE | - |
| G | PAXGENE TUBE 1 | PAXGENE RNA SAMPLE RECORD LOG SHEET **(ONLY IF STORING PAXGENE TUBES LOCALLY IN -20°C FREEZER)** |
| H | PAXGENE TUBE 2 | PAXGENE RNA SAMPLE RECORD LOG SHEET **(ONLY IF STORING PAXGENE TUBES LOCALLY IN -20°C FREEZER)** |
| J | FTA CARD PAPER COVER. (APPLY LABEL DIRECTLY OVER QIAGEN PHONE DETAILS ) | TO FRONT OF THE FTA ENVELOPE (PLACE BELOW THE FOLDING LINE INDICATED BY NOTCHES ON ENVELOPE) |
| K | SST TRANSFER TUBE | - |
| L | LITHIUM HEPARIN TRANSFER TUBE | - |
| M | 6ML EDTA TRANSFER TUBE | - |
| P | URINE TRANSFER TUBE | URINE SPECIMEN COLLECTION CUP |

.

Supplies can be ordered from Angela Rinaldi in the SERA Glasgow Biorepository using the ‘Clinic and Lab Consumables Order Form.xls’ which can be downloaded from the SERA e-CRF Documents page.

E-mail: angela.rinaldi@glasgow.ac.uk.

Tel : 0141 211 1759

**Sample Collection**

If at all possible, samples should be taken in the morning to allow samples to be transferred and processed on the same day. Those samples taken at remote sites must be shipped using a next day pre 1200 delivery service.

Ensure each sample collection bottle is completely filled to give maximum yield, prevent variation in the concentration of the gel (where applicable) and for efficient separation.

**BDP100 and Paxgene RNA Tubes**

Ensure that the PAXgene RNA tubes are at room temperature prior to use. If for any reason the PAXgene RNA tube is the only tube to be drawn, blood should be drawn into a ‘discard tube’ prior to drawing blood into the PAXgene tube.

Both the BDP100 and Paxgene blood tubes contain a chemical additive, it is important to avoid possible backflow from the tube thereby avoiding any possible adverse donor reactions. For all blood collections to guard against backflow, observe the following precautions:

1. Place donor's arm in a downward position.

2. Hold tube with the stopper uppermost.

**3. Release tourniquet as soon as blood starts to flow into tube.**

4. Allow vacuum to be exhausted before removing tube.

5. Make sure tube additives do not touch stopper or end of the needle during venepuncture.

### 2.2.2 Blood draw order

The sample tubes contain additives. It is important to follow the correct order of draw to prevent contamination of samples, so samples should be taken in the following order:

1. SST tube

2. Lithium/Heparin Tube

3. 6ml EDTA tube and 10 ml EDTA Tube for DNA.

4. BDP100 Tube

5. PAXgene RNA tube

If insufficient bleed to fill all of tubes then 10ml EDTA DNA tube may be taken at 6 or 12 month visit.

### 2.2.3 Collection of mid-stream specimen of urine

Apply the relevant barcode label to the blue topped urine collection pot and give to the patient. Inform the patient that the collection cup lid has a recessed needle in the cap which is covered by a sticky label and NOT to remove the label as there is a risk of needlestick injury.

Ask the patient to wash their hands before and after urine collection. Instruct how to collect the sample

1. Removing the lid from urine collection cup
2. Voiding a small amount of urine into toilet then stopping mid-stream.
3. Continuing to pass urine into the collection cup until at least half full.
4. Replacing the lid of the urine collection cup ensure it is screwed on tightly.

## 2.3 Sample labeling

### 2.3.1 Bar codes

All sample tubes and transfer tubes are to be labeled using bar codes from the sample pack according to Table 1 above. Apply the barcode labels in a lengthwise orientation. For the SST, Lithium Heparin and 6ml EDTA tubes, apply the barcode label over the blood tube label to allow a good view of the interface between the cells and supernatant when pipetting samples. For transfer tubes, apply the barcode labels over the frosted area of the tube to allow laboratory staff better view of tube contents.

At the Central Processing Centre, a barcode reader will be used to link the samples to the patient ID – the nurse on site need only ensure that samples are drawn in the appropriate order and samples are decanted into the correct transfer bottle.

## 2.4 Precautions

### 2.4.1 PAXgene RNA tubes

Should always be withdrawn last. Follow manufacturers instructions when withdrawing blood.

### 2.4.2 Urine Collection Pots

The urine collection cup lid has a recessed needle which is covered by a sticky label. Advise the patient not to remove the label as this puts them at risks of needlestick injury.

# PRELIMINARY SAMPLE PROCESSING ON-SITE

## 3.1 Equipment list

1 x Centrifuge with swing-out rotor or

1 x Centrifuge with 45 degree fixed-angle rotor

Small ice bucket and chilled gel packs

Wire rack x 2

Domestic fridge with freezer compartment or -20^0^C freezer

Transport specimen boxes

- Room temperature transport boxes
- 4^0^ C temperature transport boxes
- -20^0^ C temperature transport boxes
- Sample record log sheet

## 3.2 Preliminary processing procedures

Some preliminary processing will be required on all sites. Some recruitment sites will be located close to a central processing laboratory and able to transfer blood tubes within 2 hours of removal. These sites will transfer the PAXgene RNA tubes at room temperature. The preliminary sample processing for each tube is as follows:

### 3.2.1 SST tubes

- immediately after collection, gently mix the blood by inverting the tube 5 times.
- stand tube upright and keep at room temperature for 30-60 minutes prior to centrifuging to ensure clot formation
- centrifuge blood samples for 10 minutes on a swing-out rotor (or 15 minutes on a 45 degree fixed-angle rotor) at 1200g.
- apply the SST transfer tube barcode label to the recipient tube
- carefully collect the fluid layer with an appropriate transfer pipette without disturbing the cell pellet and place this into a labeled recipient tube . Ensure that the recipient tube is clearly marked as recipient for SST tube.
- place all samples at a temperature of 4^o^C with the chilled gel pack, until they are ready for dispatch to the central processing laboratory in the provided pre-prepared sample box still with the gel packs to retain the low temperature **ON THE SAME DAY.**

### 3.2.2 Lithium Heparin, 6ml EDTA and BDP100 tubes

- immediately after collection, gently mix the blood by inverting the tube 10 times.
- place tube in wet ice before centrifuging, which should be done within 30 mins of collection.
- centrifuge blood samples for 10 minutes on a swing-out rotor (or 15 minutes on a 45 degree fixed-angle rotor) at 1200g.
- apply the Lithium Heparin and 6ml EDTA transfer barcode labels to the recipient tubes
- BDP100 tube require no further processing.
- **For Lithium Heparin and 6ml EDTA only:**
- carefully collect the fluid layer with an appropriate transfer pipette without disturbing the cell pellet.
- DO NOT TAKE ALL THE PLASMA. **Collection of all the plasma may cause contamination of the underlying buffy coat and red blood layer cells. Leave approximately 0.5cm of plasma to avoid collection of underlying buffy or red blood cells.**
- place supernatants into the appropriately labeled recipient tube.
- Ensure that the recipient tubes are clearly marked as recipient tube for either Lithium Heparin or 6ml EDTA.
- place all samples at a temperature of 4^o^C with the chilled gel packs, until they are ready for dispatch to the central processing laboratory in the provided pre-prepared sample box still with the gel packs to retain the low temperature **ON THE SAME DAY.**

### 3.2.3 PAXgene RNA

- immediately after collection, gently mix the blood by inverting the tube 10 times.
- samples need to be stored at room temperature for EXACTLY two hours
- if the samples will be transferred to the central processing laboratory within two hours of sampling, then no further processing is required

otherwise, after two hours, place the Paxgene tubes in a wire rack and place in a -20^o^C freezer for at least 24 hours.

- Samples should be held in-site until they are ready for dispatch to the central processing laboratory in a temperature-controlled shipping box in batches**.**
- Paxgenes which are stored at -20^o^C are stable at this temperature for at least 4 years. **Dry ice collections will be arranged either on a quarterly basis or upon approaching local freezer capacity.**

### 3.2.4 10ml EDTA tube for DNA

- immediately after collection, gently mix the blood by inverting the tube 10 times.
- place tube directly into transfer box for subsequent processing at Central Laboratory.

### 3.2.5 QIAcard FTA One Spot

- WEAR GLOVES TO AVOID CONTAMINATING THE SAMPLE AREA.
- Prepare the FTA card by opening it up to expose the sample area.
- Immediately after withdrawing the needle from the patients arm place the tip of the needle onto the sample area and spot blood onto the card.
- Avoid puddling and use a concentric circular motion to place the sample onto the card.
- Do not rub or smear the sample onto the QIAcard.
- Allow the QIAcard to dry for one hour at RT and then close cover but do not seal.
- Place QIAcard in provided plastic bag and place in chilled box with other samples for transport.

### 3.2.6 Urine Specimen

- Transfer the urine from the collection pot into the brown topped Vacutainer tube using

the integrated transfer needle.

- The urine samples can be centrifuged at the same time and speed as the other SERA

samples. 30 mins and 1200g.

- After centrifuging, using a 5ml syringe and white needle, withdraw the urine up into the

syringe taking pulling th e syringe pluger as far back to take > 5mls urine.

- Remove the needle and attach the 0.2micron filter to the luer slip end and place the leur

lok end of the filter in to the opening of the urine transfer tube.

- Depress the plunger and filter at least four mls of urine into the transfer tube.
  1. ***Sample Record Log***Complete the Sample Record log, recording the number of tubes collected and processed.
     It is important to accurately note on the record sheet the time when blood samples were taken, processed and dispatched.

# DISPATCH OF SAMPLES TO CENTRALISED LAB

## 4.1 Transportation (see Appendix 1)

### 4.1.1 Research Nurse (within 2 hours)

Where the research nurse taking the sample is able to transport the samples directly to the Central Processing Laboratory within 2 hours, they should take all the spun samples in a chilled temperature-controlled box, but the PAXgene samples should be taken at room temperature.

### 4.1.2 Research Nurse (same day, but >2 hours)

If the research nurse cannot get all samples to the Central Processing Laboratory within two hours, the SST, Lithium/Heparin, EDTA and BDP100 samples should be taken in the provided chilled temperature-controlled box. The PAXgene samples should be placed upright in a wire rack in a -20^0^C freezer on site, and transferred in batches every month by commercial courier.

### 4.1.3 Commercial transport – chilled, same day

If the research nurse who takes the samples is not in a position to transfer the samples to the Central Processing Laboratory, the SST, Lithium/Heparin, EDTA and BDP100 samples should be processed as above, stored and then transported in the provided chilled temperature-controlled box **ON THE SAME DAY.** The administrative details for the commercial transport arrangements will be provide with the pre-packed box.

### 4.1.4 Commercial transport – frozen (on dry ice) in batches every month

If PAXgene samples cannot be transferred to the Central Processing Laboratory within two hours, then the samples should be stored on site at -20^0^C and transferred in batches **EVERY MONTH.**

***4.2 Arranging and recording transfer of samples***

Notify the relevant central processing laboratory that transport has been requested. Before transfer, remember to include the updated Sample Record logs to the boxes. After collection, contact the central processing laboratory to notify of dispatch.

### 4.1.4 Sample Record log

An example of the Sample Record logs is shown in Appendix 2. It records:

- Centre
- Patient study number
- Sample types collected
- day/month/year of sample collected
- time of sample collections (24hour clock)
- printed name of person responsible for sample processing & storage
- signature of person responsible for sample processing & storage

# FULL SAMPLE PROCESSING AT CENTRAL LABORATORIES

When the vacutainers arrive at the central laboratory, they will be processed as soon as possible according to Table 5.1. All of the vacutainers that arrive will be scanned and entered into the LIMS system using a barcode reader.

| **Vacutainer Tube** | **Fractions** | **Number of Aliquots** |
| --- | --- | --- |
| SST Tube | Serum | 4 |
| Lithium/Heparin Tube | Plasma | 4 |
| 6 ml EDTA | Plasma | 4 |
|  | Buffy | 1 |
| BDP100 | Plasma | 10 |
| Urine | Urine | 4 |
| **TOTAL ALIQUOTS** |  | 27 |

**Table 5.1 Fractions and aliquots of blood samples**

Sample processing and LIMS operations are covered by LAB_SOPS 001 to 007 and LIMS User Manual Version 0.2

# DEPARTURES FROM SOP

It is acknowledged that resources or staffing levels will vary and may not permit the implementation of all of the protocol listed in this SOP, just as not all blood draws will yield adequate volumes required to store in the aliquots recommended. It therefore becomes the responsibility and discretion of the individual(s) processing the sample to optimize as closely as possible to the recommended storage volumes. In instances where the SOP cannot be followed it is important that blood-processing variables be noted on the Sample Record log.

**APPENDIX 1**

**Make patient appointment**

If possible, arrange for your patients to be seen in the morning, to allow for same day transfer and processing of specimens. If this is not possible, samples can be sent chilled, overnight.

**Book sample collection***

**Collect samples from patient**

**Spin Lithium/Heparin, 6 ml and 10 ml EDTA, BDP100 and urine samples immediately or at least within 30 mins**

**Filter urine using a 0.2micron filter into a 4ml transfer tube**

**Decant serum/plasma from Lithium/Heparin and 6ml EDTA tube into labeled transport bottles**

Place in a wire rack on ice in a chilled, temperature-controlled box

**Allow SST sample to stand for 30-60 minutes at room temperature before spinning**

**Decant serum from SST tube into labeled transport bottle**

Place in a wire rack on ice in a chilled, temperature-controlled box

**Complete Sample Record Log and dispatch sample**

**Inform Central Processing Laboratory**

**Leave Paxgene RNA sample for 2 hours at room temperature**

**Freeze Paxgene RNA at -20^0^C**

**Quarterly or once freezer approaches capacity,**

**transfer frozen samples on dry ice**

* if using a commercial courier.

| **Centre** |
| --- |
| 01 |

**Appendix 2A**

**Paxgene RNA Sample Record Log**

| **STUDY NUMBER** | **CHI NUMBER** | **PAXGENE 1BARCODE LABEL** | **PAXGENE 2BARCODE LABEL** | **DATE COLLECTED** | **TIME COLLECTED** | **TIME FROZEN** |
| --- | --- | --- | --- | --- | --- | --- |
|  |  |  |  |  |  |  |
|  |  |  |  |  |  |  |
|  |  |  |  |  |  |  |
|  |  |  |  |  |  |  |
|  |  |  |  |  |  |  |

**Dispatch all samples on dry ice in chilled transport box by commercial courier.**

| **Date of collection** | **Print:** |
| --- | --- |
| **Time of collection** | **Signature:** |

**Appendix 2B**

| **RECORD LOG LABEL** |
| --- |
|  |

**Sample Record Log**

**SST/LITHIUM HEPARIN/EDTA/BDP100/PAXGENE RNA/FTA CARD/URINE/SYNOVIAL FLUID**

| **Centre** | **Study Number** | **CHI number** |
| --- | --- | --- |
|  |  |  |

**1. Samples Collected: Insert either Y/N (yes/no).**

| SST | Lith Hep | 6ml EDTA | 10ml EDTA  for DNA | BDP100 | Paxgene RNA 1 | Paxgene RNA 2 | FTA CARD | Urine | Synovial Fluid |
| --- | --- | --- | --- | --- | --- | --- | --- | --- | --- |
|  |  |  |  |  |  |  |  |  |  |

| Sample Collection: | Time: |
| --- | --- |
|  | Date: |

**2. Within 30 minutes 🡺 spin the Lith Hep, 6ml EDTA, Urine and BDP100 tubes for 15mins**

| Time centrifugation started: |  |
| --- | --- |

**3. Leave SST tube for 30-60mins at room temperature and then spin for 15 mins**

| Time centrifugation started: |  |
| --- | --- |

**4. If the samples will not reach the biobank within two hours, transfer the Paxgene RNA bottles to a -20^0^C freezer after two hours exactly, and fill in the Paxgene RNA storage log.**

| Paxgene RNA samples included: | Yes / No |
| --- | --- |

**5. Dispatch samples in chilled transport box on the same day.**

| Time of collection | Print name: |
| --- | --- |
|  | Signature: |

**Central Processing Laboratories**

The contact details for the Central Processing Laboratories are as follows:

**Glasgow**

SERA samples from the following study sites will be sent to the NHS GGC Biorepository lab for processing and storage.

**NHS Greater Glasgow and Clyde (local)**

- Gartnavel General Hospital
- Glasgow Royal Infirmary
- Stobhill Hospital
- Victoria Infirmary,
- Southern General Hospital
- Royal Alexandra Hospital
- Vale of Leven,
- Inverclyde Royal Hospital

**NHS Lanarkshire (local)**

- Wishaw General Hospital,
- Hairmyres Hospital,
- Monklands General Hospital,

**NHS GGC Address:**

NHSGGC Biorepository

C/O Glasgow CRF

Tennent Institute

38 Church Street

Western Infirmary

Dumbarton Road

Glasgow

G11 6NT

**Tel:** 0141 211 1759

**SERA Technician:** Angela Rinaldi

Angela.rinaldi@glasgow.ac.uk

**Remote sites**

- Heathfield Clinic, Ayr,
- Dumfries and Galloway Royal Infirmary
- Victoria Hospital, Kirkcaldy
- Perth Royal Infirmary
- Ninewells Hospital, Dundee
- Raigmore Hospital, Inverness
- Aberdeen Royal Infirmary

.

**Edinburgh**

Samples from Borders General Hospital, and WTCRF, Edinburgh will be processed and stored at NHS Lothian Biorepository lab

**Address**:

2nd Floor, Molecular Medicine Centre, Western General Hospital, Crewe Road, Edinburgh, EH4 2XU

**Tel:** 0131 651 1028

**SERA Technician:** Dil Kalbir [v1dkabir@staffmail.ed.ac.uk](mailto:v1dkabir@staffmail.ed.ac.uk)
