# Supplementary material for: The Scottish Early Rheumatoid Arthritis (SERA) Study: an inception cohort and biobank
Source: BMC Musculoskelet Disord. 2016 Nov 9;17:461. doi: 10.1186/s12891-016-1318-y (PMC5103386; doi:10.1186/s12891-016-1318-y)
Supplement: Additional file 3: — SERA Access Policy Holdings. (DOC 46 kb) [file 12891_2016_1318_MOESM3_ESM.doc]

**Scottish Early Rheumatoid Arthritis Inception**

**Cohort and Biobank**

*Access Policy*

**Contents**

**Summary ............................................................................................................................3**

**Objective of the Access Procedures .................................................................................4**

**Access charges....................................................................................................................5**

**Confidentiality ...................................................................................................................6**

**Managing Access to SERA resources..............................................................................7**

**Summary**

The Scottish Early Rheumatoid Arthritis inception cohort and biobank (SERA) is a collaborative project between NHS Scotland, Scottish academics, Pfizer Inc and the Chief Scientist’s Office. It will recruit participants with newly diagnosed rheumatoid arthritis (RA) or undifferentiated arthritis (UA) who will give their consent, answer questions, undergo physical examination and give samples (blood, urine and synovial fluid) at baseline and follow up assessment visits. The data and samples collected will together constitute an important resource (‘the Resource’) for future scientific study.

SERA’s objective is to encourage the extensive and appropriate use of the Resource for research. The aim of these Access Procedures has been to ensure that they facilitate ethical research, in line with the undertakings given to the participants when they agreed to take part. The procedures recognise the rights of Pfizer to protect their commercial interests. The procedures and processes that have been applied to access to the Resource derive from the following key principles:

- All applications to use the Resource will be checked to ensure that they are consistent with these Access Procedures and the consent that was provided by the participants.
- The Resource is available to all bona fide academic researchers for all types of health-related research that is in the public interest, without preferential or exclusive access for any person. All researchers, whether in universities, charities, or government agencies, and whether based in the UK or abroad, will be subject to the same application process and approval criteria.
- Access to the Resource by commercial companies will only be possible through partnership with an academic partner. Pfizer Inc will have the right to veto the use of biological samples by commercial companies until April 2015, but agreement will not be unreasonably withheld for projects of scientific and public interest where there is no conflict with the commercial interests of the company, and no transfer of IP rights to another company.
- Access to the biological samples that are a limited and finite resource will be carefully controlled and coordinated. The quantity of sample that is required will be judged against the potential benefits of the research project, with advice from appropriate experts when required.  SERA Access Procedures v1, 17/05/2012 3
- Safeguards will be maintained to ensure the anonymity and confidentiality of participants’ data and samples. Researchers will enter into a legal agreement not to make any attempt to identify participants, and the data and/or samples provided by SERA to researchers will not identify any particular participant (i.e. they will be “anonymised”).
- External applicants will be expected to pay for access to the Resource on a cost-recovery basis, with a fixed charge for managing the application review process and a variable charge depending on how many samples, tests and/or data are required for the approved research project.
- SERA will remain the owner of the database and samples, but will have no claim over any inventions that are developed by external researchers using the Resource.

**Objective of the Access Procedures**

- 1. The objective of these Access Procedures is to facilitate access to the samples and data so that they get the widest possible usage while ensuring that such access and usage is consistent with the undertaking given to the participants (see below) and the wider public interest (including being lawful and compatible with respect for human rights).
  2. At all times, decisions to grant access should maintain the undertakings made to participants when they consented to take part: (i) to ensure that any uses of the resource are consistent with its stated aims; (ii) to maintain participants’ anonymity and confidentiality (as far as reasonably possible).
  3. It is intended that these Access Procedures are clear and transparent and are implemented in a manner which is proportionate, accountable and fair. The Procedures provide a framework for addressing and determining access issues. They deliberately do not prescribe what will be done in each and every circumstance on the basis that SERA cannot (and would be unwise to try to) predict the nature of access requests over the long-term. The procedures may be amended, as required, by the SERA Scientific Steering Committee.
  4. To protect the commercial interests of Pfizer for the duration of the project (or beyond if Pfizer continues to be the sole commercial funder for SERA), third party commercial involvement with the Resource will only be approved with the explicit written agreement of Pfizer Inc, who will have a right to veto that involvement. However, Pfizer undertakes that consent will not be unreasonably withheld for projects of scientific and public interest where there is no conflict with the commercial interests of the company.

**Access charges**

1. SERA has carefully considered a variety of charging models and has decided to adopt the following approach:
   1. Pfizer/SERA investigators will not be charged access fees
   2. All external researchers are to be charged on the same cost-recovery basis;
   3. There is a fixed charge for each Application of £2,000 (plus VAT if applicable) to cover  SERA’s costs for the administration of the Application. This charge is payable as £250 on submission of the Preliminary Application Form and £1,750 on submission of the Main Application Form; and
   4. There is a variable component which depends on what is being accessed, calculated either as a simple cost recharge of SERA’s estimated internal costs or as a recharge of third party costs incurred by SERA. This variable component will include charges for sample extraction, sample assays, re-contact costs, data derivation and (for large data sets) data extraction.
2. SERA will keep this charging policy under review (including the possibility of different levels of charge for different types of user) to ensure that it continues to represent an equitable, balanced and pragmatic approach

**Confidentiality**

1. Each person who consented to join the SERA project received a clear assurance that all of their personal information would be held in strict confidence with careful safeguards, and that no identifiable information about any participant would be available to anyone outside of SERA. Although SERA cannot guarantee that the identity of participants will remain confidential in all circumstances, it uses such technical and legal measures that are available to it to ensure that (as far as possible) each participant’s identity remains confidential.
2. Identifying information is retained by SERA to allow it to make contact with participants when required and to link with their health-related records. The level of access that is allowed to staff within SERA is controlled by unique user names and passwords, and restricted on the basis of their need to carry out particular duties.

1. SERA will aim to make as much data as possible publicly available in grouped format (i.e. not at individual participant level) on its website, or through collaboration with INBANK, so that potential research users can evaluate what the Resource contains before applying to use it. This includes a list of the questionnaire and measurement data variables and types of biological samples collected at the baseline assessment, along with the numbers of participants in various groupings of the data variables.
2. SERA will require researchers to make a formal application for data and/or samples relating to selected participants (e.g. based on information from the baseline assessment or subsequent follow-up of their health outcomes).
3. When data are provided to researchers, every effort will be made to remove both direct and indirect markers which might allow the identification of an individual. The Material Transfer Agreement will include a strict prohibition on researchers trying to identify any participant. In the case of inadvertent identification, researchers are required to report this immediately to SERA and make no attempt to contact the individual.

**Managing Access to SERA resources**

1. Decisions about granting access to SERA resources will be made by the Scientific Steering Committee who will apply a standard set of criteria (subject to ongoing review and amendment by SERA Scientific Steering Committee) to the assessment of all Applications:
   1. compatibility of the research project with the purposes of SERA, which is to further knowledge about and treatment of rheumatoid arthritis, undifferentiated arthritis and other rheumatic diseases;
   2. clinical and scientific relevance of the research question to be addressed;
   3. the feasibility of the Research Project, and demonstrable technical capability to deliver the same;
   4. whether the project will utilize a significant amount of the finite samples.
2. Decisions will be taken by majority vote of the Scientific Steering Committee, except in cases where the application is from a commercial company, when Pfizer Inc will have sole discretion about granting access to the Resource. Where the SERA academic partners believe that Pfizer Inc. has been unreasonable in withholding consent, the matter will be referred to the TMRC2 Steering Committee who will provide binding arbitration.
3. Access to samples/data will be granted to academic, charitable, governmental or commercial organizations according to the following guidelines:
   1. academic, charitable and governmental applicants will normally be granted access to the Resource, if the proposed project is of sufficient scientific merit, subject the payment of Access costs (if applicable), and completion of a Material Transfer Agreement (if required).
   2. academic collaborations with external funding from commercial companies will be viewed favourably where the proposed project is of sufficient scientific merit, subject to the payment of Access costs, and completion of a Material Transfer Agreement (if required) **AND** where there is no conflict with the commercial interests of Pfizer Inc.
   3. UK or multi-national consortia between academic centres and multiple industrial partners will be able to apply for access to the Resource, and such applications may be granted where the proposed project is of sufficient scientific merit, subject the payment of Access costs, and completion of a Material Transfer Agreement (if required) **AND** where there is no conflict with the commercial interests of Pfizer Inc.
   4. third party commercial companies will not be granted access to the Resource, without the explicit consent of Pfizer Inc who will have sole discretion to veto such applications, for the duration of the project within the time defined above
4. Where Pfizer Inc perceives there to be a conflict with their commercial interests, no specific reason need be given for denying an application; where possible, however, such an explanation would be provided in the interests of partnership and transparency.
5. There are two stages to the application process:
6. *Preliminary/informal enquiries* to the Scientific Steering Committee are welcomed, to allow researchers to determine: (i) whether their proposed research use is likely to be approved; (ii) whether the resource contains the data and/or samples required for their proposed research; and (iii) the indicative cost of obtaining such data and/or samples (e.g. in preparation for a funding application).
7. *Formal Application submission –* a formal application may be subsequently submitted and will include:
   1. Lay summary (200 words or less) of the Research Project;
   2. Study protocol
   3. Required data and/or quantity and type of samples, with justification;
   4. Details of the scientific rationale, protocol, feasibility, members of the research team, and funding.
8. A web based standard application form will be provided to assist submission and review. The Scientific Steering Committee may ask the applicants for further details as required, in order that the committee can judge (i) whether the proposed research use meets the required criteria for access (including legal and ethics standards); (ii) whether the amount of sample required is scientifically justified; and (iii) the cost of providing such data and/or samples.
9. *Material Transfer Agreement (MTA):* For approved applications, the Material Transfer Agreement will need to be executed and access charges paid before release of data and/or samples to the applicant.

5. Timescale – web-based preliminary applications can be submitted at any time, and will be assessed by a member of the local management team. Subsequent formal applications will be reviewed by the Scientific Steering Committee within 6 weeks of receipt.
